# 

**Published:** 1890-02

**Authors:** 


### CONTENTS—FEBRUARY, 1890.—VOL. 5, NO. 8.

ORIGINAL COMMUNICATIONS— Page
Life; an Essay. W. A. Morris, M. D . . . . . . . . . 280
Paretic Dementia; Discussion before the Medico-Legal Society, of Chicago . . . . . . . . . . . . . . . 287
The Microscope. F. E. Yoakum, M. D. . . . . . . . 296
Potassii Nitras in Chill and Fever. J. D. Hunter, M. D., New Orleans, La. . . . . . . . . . . . . . . . . . 300
CORRESPONDENCE—
Letter from Dr. Lowry; His Case of Extra-Uterine Pregnancy. . . . . . . . . . . . . . . . . . . . . . . 303
Letter from Dr. C. M. Rosser . . . . . . . . . . . . 305
Letter from Dr. Perry; Gastro-Intestinal Catarrh. . . . 306
Letter from Dr. R. G. Williams; Sweating Feat . . . . 308
SOCIETY NOTES—
Texas State Medical Association; Circular Letter from the President . . . . . . . . . . . . . . . . . . . 308
Travis County Medical Society; Proceedings . . . . . 309
Austin District Medical Society . . . . . . . . . . . 310
Tri-State Sanitary Association . . . . . . . . . . . . 310
EDITORIAL—
Official Preparations of Uncertain Strength . . . . . . 311
MEDICAL NEWS AND MISCELLANY—
Numerous Items . . . . . . . . . . . . . . . . . . . 315
PUBLISHER'S NOTES . . . . . . . . . . . . . . . . . 319

